# Out-of-Plane Behavior of Masonry Prisms Retrofitted with Shape Memory Alloy Stripes: Numerical and Parametric Analysis

**DOI:** 10.3390/s22208004

**Published:** 2022-10-20

**Authors:** Alireza Tabrizikahou, Mieczysław Kuczma, Magdalena Łasecka-Plura

**Affiliations:** 1 Institute of Building Engineering, Poznan University of Technology, Piotrowo 5, 60-965 Poznan, Poland; 2 Institute of Structural Analysis, Poznan University of Technology, Piotrowo 5, 60-965 Poznan, Poland

**Keywords:** masonry, out-of-plane, shape memory alloy, retrofitting, strengthening, finite element, Abaqus

## Abstract

This paper provides a novel Finite Element (FE) simulation to estimate the out-of-plane response of masonry prisms retrofitted with Shape Memory Alloy (SMA) stripes. Empirical data were utilized to develop the computational analysis parameters (mechanical parameters for brick, mortar, and SMA materials) as well as the calibration of the computational FE-based models. For this purpose, a complete micro-modeling approach was applied, assuming perfect contact between mortar joints and brick units. A Concrete Damage Plasticity (CDP) model was developed to define the constitutive relation between brick and mortar. SMA stripes were mortar-installed on the surface of the prisms with a perfect connection. The masonry prism’s verified computational model was utilized to generate parametric research to explore the effect of varying SMA stripe thicknesses and different SMA usage (Ni-Ti or Cu-Zn-Al). The FE study findings indicated that, independent of their material type or thickness, using SMA stripes greatly minimizes brick prism deterioration. SMA stripes greatly decreased residual displacement and plastic strains. Parametric tests, however, revealed that employing Ni-Ti SMA and increasing its thickness is more effective with respect to the masonry prism out-of-plane response than Cu-Zn-Al SMA.

## 1. Introduction

Construction catastrophes are frequently the result of inadequate element size, material characteristics, and structural flexibility, which together result in a lack of resistance. Brittle construction materials, such as unreinforced concrete and unreinforced masonry (URM), exhibit limited ductility when subjected to severe loadings.

According to the literature, URM walls transfer interior stresses that develop within structural elements to neighbouring members or foundations [1,2]. Out-of-plane loadings, e.g., those induced by earthquakes and wind, make URM extremely susceptible to being used as a load-bearing component of a structural system due to its low load-carrying capacity and ductility [3,4,5]. Engineers have therefore determined that structural retrofitting of URM is a crucial issue that has to be addressed.

Common reinforcing techniques might not be sufficient to provide significant resilience to the highest expected seismicity [6,7]. Using these technologies to strengthen brick walls is often challenging because of increased construction weight, financial requirements, poor performance at high heat, and difficulties with adhesion to the surface of brick walls [8,9].

Extensive research has been conducted to improve the out-of-plane behavior of URM walls. Typically, this is carried out by retrofitting components with superior structural performance to the wall’s surface [10,11,12,13,14]. Additionally, cement or concrete is frequently used as an adhesive component in the installation of such retrofit components. Dowels or epoxy can be used to apply other materials, such as fiber-reinforced polymers (FRPs), to the walls [15,16].

All of the aforementioned studies have comprehensively investigated several retrofitting methods, the majority of which are based on the use of conventional materials (e.g., concrete and steel) in various forms, such as shotcrete, steel-striping, etc. Other studies have investigated the application of FRPs in strengthening URM walls. The use of smart materials in strengthening URM walls against out-of-plane loadings has not been fully studied, and this seems to be a distinctive component of the current research.

A unique method for retrofitting URM walls is the use of smart materials such as Shape Memory Alloys (SMAs). SMAs can return to their original shape following severe deformation, which is one of their many distinctive and advantageous qualities. This results from a martensitic phase change brought on by either heating (the shape memory effect) or removing the applied tension (pseudoelasticity) [17,18,19].

To assess the usage of SMAs for retrofitting masonry structures, a considerable amount of research has been carried out both empirically and numerically. A few of these investigations and their findings are discussed in this study.

Casciati and Hamdaoui [20] examined the utilization of pre-tensioned SMA wire-based linked equipment in the refurbishment of old masonry structures. Their empirical research findings were utilized to create a computational model to account for the effects of SMA equipment. The structure was initially examined in its original form without retrofitting treatments applied, then the effects of various retrofitting processes were confirmed empirically and integrated into the quantitative simulation by altering the equivalent Young’s modulus accordingly.

Cardone et al. [21] devised a method for enhancing the seismic behavior of steel-based joints in historic structures using pre-tensioned SMA wires. The results of their tests revealed that the proposed method is efficient in decreasing force disparities induced by temperature fluctuations in the environment. The authors suggested that the studied SMA-based device was highly beneficial in optimizing the thermodynamic behavior of steel joints. Moreover, force variations in steel connectors induced by changes in air temperature were reduced by 80–90 percent using SMAs.

Habieb et al. [22] examined the use of hybrid SMA-based wires with a fastened fiber-reinforced rubber isolator as the foundation system for a historic cathedral’s seismic resilience modofication. The suggested approach, using a 2% pre-strained SMA cable, demonstrated the largest reduction of the church’s lateral deflection, for considerable damage minimization in the case of large seismic events, thanks to its strong energy dissipation capacities.

Rezapour et al. [23] numerically investigated the in-plane behavior of URM walls retrofitted with post-tensioned SMA strips in two different configurations of crossovers and parallels and distances from each other. According to the results of this investigation, the stiffness rose by 98.1 percent in the case of vertical strips mounted to the walls and by 127.9 percent in the case of crossover placement. Moreover, while the maximum resistance in the parallel arrangement was 108 kN, by the end of cyclic loading it had decreased by more than half to 40 kN.

Tabrizikahou et al. [24] numerically analyzed the application of a novel hybrid retrofitting method using SMA stripes embedded in engineered cementitious composite (ECC) installed on masonry brick walls. A parametric analysis was carried out to investigate the effect of different retrofitting factors on the cyclic behavior of the masonry walls. According to the numerical results, the total energy absorption in the hybrid SMA-ECC retrofitting system rose by 318 percent compared to URM. In addition, the hybrid model produced a maximum reaction force of about 246 kN.

To evaluate the use of SMAs to enhance the out-of-plane performance of masonry prisms, the current work presents finite element analyses of several masonry prisms reinforced with SMA strips mounted on the prism. The models were subjected to out-of-plane loads and their behavior was assessed.

## 2. Description of the Finite Element Models

**Figure 1:**
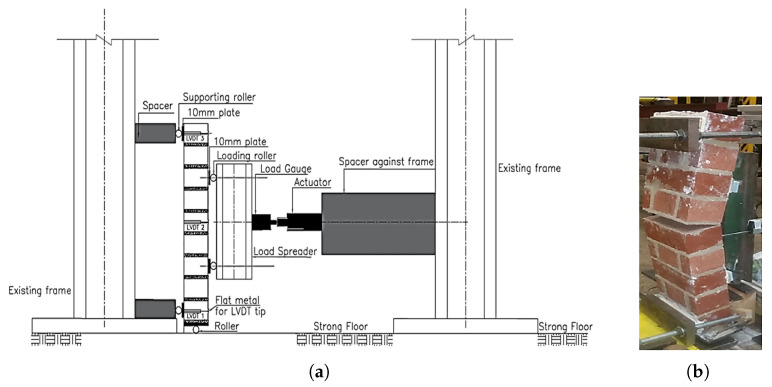
(**a**) The setup for the masonry prisms and (**b**) observed experimental failure (Reprinted with permission from [25]).

Four-point bending experiments were used to determine the out-of-plane strength of a masonry prism for perpendicular flexing, as shown in Figure 1a. A roller bearing was installed at the bottom to enable it to smoothly revolve around its foundation while bending out-of-plane. As a result, despite the perpendicular placement of the masonry prism, the experiments were carried out under braced boundary conditions and without perpendicular pre-compression stress. The test was carried out in pressure control mode, with an initial load of 200 N variations applied per two minutes until the emergence of the first fractures. Because of its location, the deterioration process began in the middle one-bed joint, which failed completely (Figure 1b).

**Figure 2:**
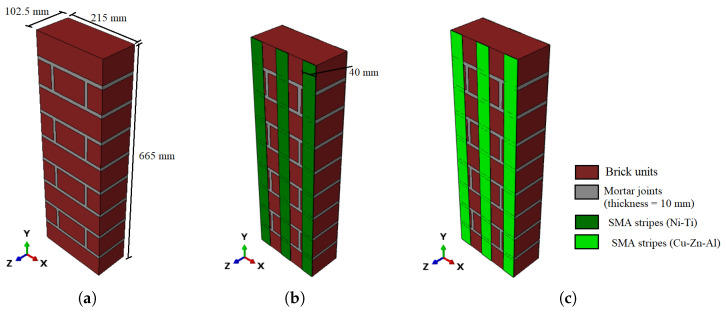
Schematic representation of the numerical model for masonry prisms with dimensions of 665 × 215 × 102.5 mm^3^: (**a**) URM prism; (**b**) masonry prism reinforced with Ni-Ti SMA stripes; (**c**) masonry prism reinforced with Cu-Zn-Al SMA stripes (SMA stripes range in thickness from 1 to 1.5 and 2 mm, respectively, with a width of 40 mm and height of 665 mm).

In this study, a computational investigation using the Finite Element Method (FEM) was carried out to gain a better understanding of SMA-based retrofitting for URM walls. We used Abaqus software [26] in our computational analyses. The primary study consisted of computational modeling of the flexural strength of a masonry prism with dimensions of 665 × 215 × 102.5 mm^3^ (Figure 2a), similar to the one experimentally tested by Dauda et al. [25]. Six SMA stripes with a width of 40 mm and the same height as the masonry prism were installed on each side of the retrofitted brick prism models (with three stripes on each side). In the parametric investigation, two types of SMA materials (Ni-Ti and Cu-Zn-Al) with thicknesses ranging from 1.0 mm to 1.5 mm and 2.0 mm, respectively, were used in computational modeling (Figure 2a,b). The next sections describe in detail the material characteristics (for brick units, mortar joints, and SMAs), interconnections, and boundary conditions.

**Figure 3:**
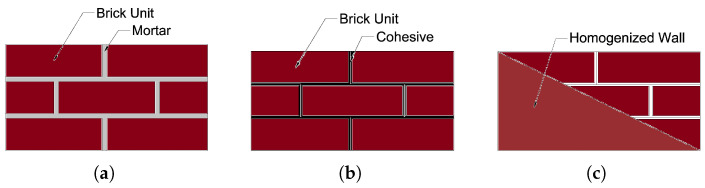
Numerical modelling methods of masonry walls: (**a**) microscopic; (**b**) simplified microscopic; (**c**) macroscopic.

There are three ways to define masonry prisms: (i) the complete microscopic technique, which thoroughly characterizes the interactions between bricks and mortar (Figure 3a); (ii) using a streamlined microscopic method, the mortar can be depicted as a coherent interaction between bricks (Figure 3b); (iii) in a macroscopic approach, the entire masonry wall is regarded as a mixture of connected and homogenous brittle materials (Figure 3c).

**Figure 4:**
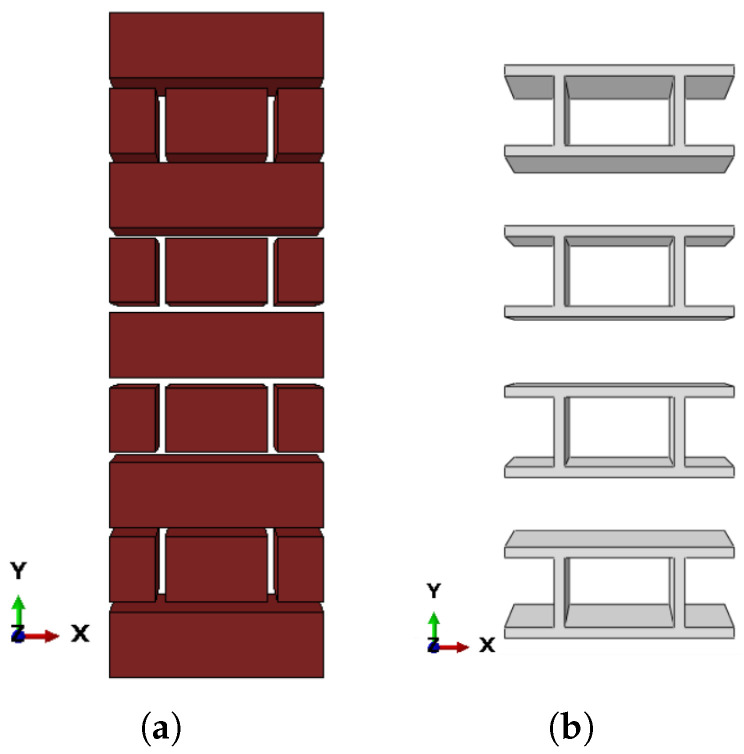
Micro-modelling approach for computational simulations: (**a**) brick units and (**b**) mortar joints.

The micro-modeling technique, in particular for small structural components in which the combined interaction of bricks and mortar is prioritized, is by far the most accurate [27]. As a result, the best method depends heavily on the exact topography of the masonry prism to be evaluated, as well as on the goals of the analysis [28]. A thorough micro-modeling approach was used in this study assuming a perfect connection between brick units and mortar, as shown in Figure 4. As a result, the interface’s mesh nodes were combined. Such a conclusion is supported by the available literature, notably the empirical experiments carried out by Iuorio et al. [29]. It is particularly well posed for insufficient mortar masonry systems, as the damage is typically influenced by mortar joints [30], and this is the subject of the present investigation.

### 2.1. Boundary Conditions and Loading

**Figure 5:**
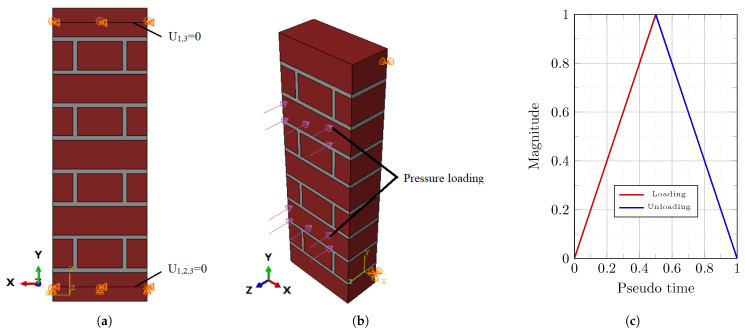
Boundary conditions in the masonry prism: (**a**) boundary condition details; (**b**) out-of-plane load application; (**c**) the amplitude of applied out-of-plane load.

The behavior of masonry walls in the event of out-of-plane force is significantly influenced by boundary conditions [31]. The masonry prism is depicted as being simply supported in the middle of the last rows of brick pieces on the top and bottom (Figure 5a). Gravitational loads and a cycle of out-of-plane exterior pressure (loading–unloading) were applied to the masonry prism model, as shown in Figure 5b. The loading–unloading cycle was used to assess the efficacy of SMA’s hysteresis characteristic on the system’s energy dissipation.

The static RIKS technique, known as the arc-length control system, was used to determine the overall response of the masonry prism enduring continuous load increase in the context of load displacement. Semi-static computational investigations, such as the testing characteristic, were carried out under load control. The load was applied proportionally throughout the study’s load stages. The equilibrium iteration ws run at each load step, and the equilibrium path was monitored in the load-displacement environment. This method is a dependable nonlinear analysis method that is able to include material damage properties in the model.

### 2.2. Interactions and Constraint Conditions

**Figure 6:**
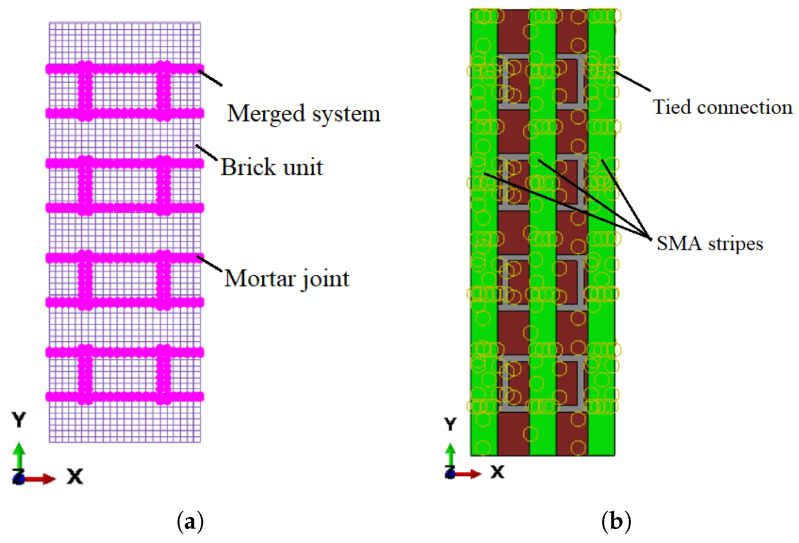
Interactions between different components of the computational model: (**a**) the masonry blocks and mortar junctions and (**b**) the masonry prism and SMA stripes.

To effectively establish a computational model, the interconnections and constraints must be adequately defined in order to accurately depict the interaction between each component of the model, namely, (i) the interface between the masonry units and the mortar joints and (ii) the interface between the masonry prism and the SMA stripes. It was assumed that the brick units and mortar joints had a perfect connection. As a result, the brick-and-mortar bonding interaction was not specified as a special interface, and the FE nodes were merged (Figure 6a).

The impact of applying different contact characteristics (tie, adhesive, surface-to-surface) is explored in another publication by the authors [24]; the contact properties between SMA stripes and masonry prism were established in the present study based on those results. An adhesive contact with tie interaction was employed to embed the SMA strips on the exterior surface of the masonry prisms. This interaction adhered the strips’ surfaces to the prism, allowing deformation and stress to be transferred from the strips to the prism (Figure 6b). The implementation of tie restrictions guarantees that, throughout the calculation, slide among SMA stripes and parallel masonry units (brick and mortar) is not allowed.

### 2.3. Constitutive Models

#### 2.3.1. Constitutive Model for Brick Units and Mortar Joints

**Figure 7:**
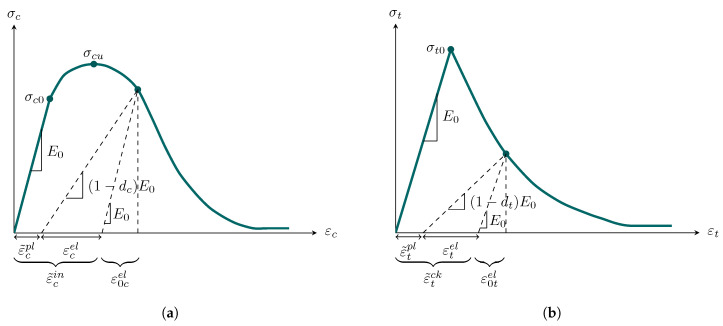
CDP modeling technique for uniaxial stress–strain relationship of semi-brittle materials: (**a**) under compression and (**b**) under tension.

Using the micro-modeling technique, the mechanical characteristics of brick units and mortar joints were considered to be isotropic and homogeneous, similar to concrete except with varying mechanical capacities. As a consequence, Lubliner’s [32] and Lee and Fenves’ [33] Concrete Damage Plasticity (CDP) model was used for computational models of semi-brittle materials. Figure 7 displays a uniaxial stress–strain relationship to demonstrate how brick units and mortar joints stiffen and degrade when exposed to compressive and tensile stresses.

The stress–strain equations for uniaxial tension and compression loads are as follows:

(1) $$\sigma_{t}=\left(1-d_{t}\right)E_{0}\left(\varepsilon_{t}-{\tilde{\varepsilon }}_{t}^{pl}\right)$$

(2) $$\sigma_{c}=\left(1-d_{c}\right)E_{0}\left(\varepsilon_{c}-{\tilde{\varepsilon }}_{c}^{pl}\right)$$

where ${\tilde{\varepsilon }}_{c}^{pl}={\tilde{\varepsilon }}_{c}^{in}-\frac{d_{c}}{\left(1-d_{c}\right)}\frac{\sigma_{c}}{E_{0}}$ and ${\tilde{\varepsilon }}_{t}^{pl}={\tilde{\varepsilon }}_{t}^{ck}-\frac{d_{t}}{\left(1-d_{t}\right)}\frac{\sigma_{t}}{E_{0}}$ are the compressive and tensile strains, respectively. In addition, ${\tilde{\varepsilon }}_{c}^{in}=\varepsilon_{c}-\varepsilon_{0c}^{el}$ and ${\tilde{\varepsilon }}_{t}^{ck}=\varepsilon_{t}-\varepsilon_{0t}^{el}$ denote the compressive hardening and tensile cracking strains, respectively, in which the compressive and tensile elastic strains can be defined by $\varepsilon_{0c}^{el}=\frac{\sigma_{c}}{E_{0}}$ and $\varepsilon_{0t}^{el}=\frac{\sigma_{t}}{E_{0}}$. Finally, the compressive and tensile uniaxial softening coefficients are defined as $d_{c}=1-\frac{\sigma_{c}}{\sigma_{cu}}$ and $d_{t}=1-\frac{\sigma_{t}}{\sigma_{t0}}$, respectively.

**Figure 8:**
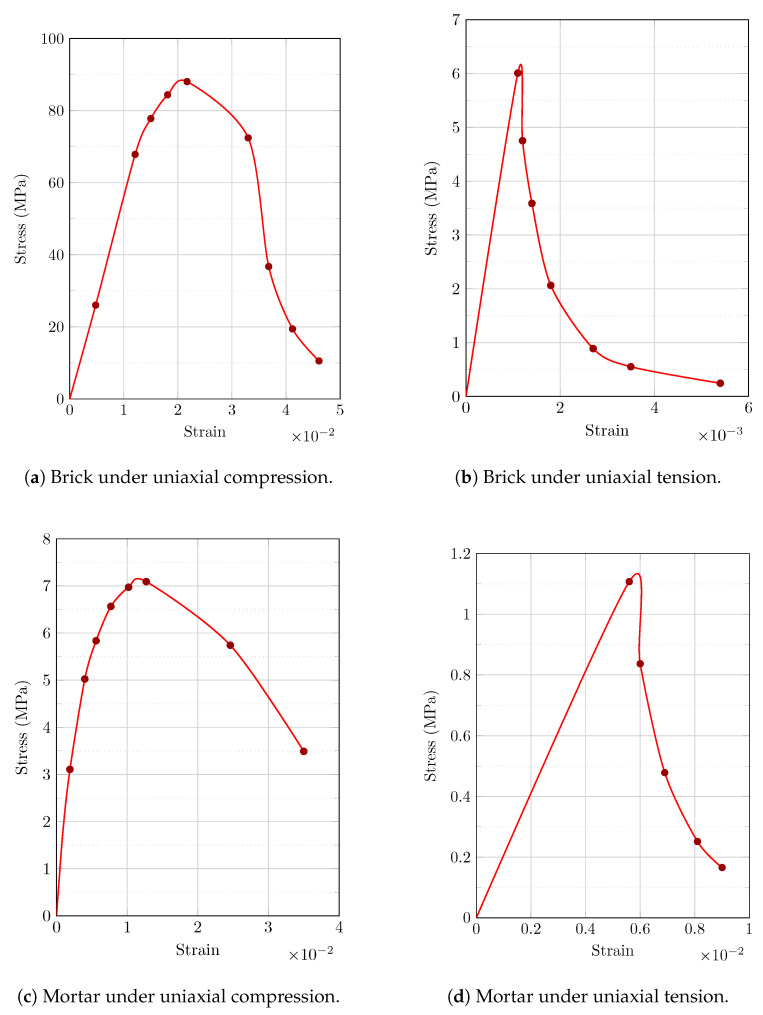
Numerical uniaxial stress–strain relationships of the brittle materials (brick units and mortar joints) used in this study: (**a**) brick under compression; (**b**) brick under tension; (**c**) mortar under compression; (**d**) mortar under tension.

Through masonry compression tests on masonry cubic specimens (215 × 215 × 215 mm^3^) together with numerical analyses, material characterization tests were conducted; the results can be found in [34,35]. The primary goal of these investigations was to obtain pertinent data on the material properties to be employed hereinafter. The Poisson ratio, Young’s modulus, and compressive strength values for both masonry components (brick units and mortar joints) were calculated, with the results shown in Figure 8 as a stress–strain graph. According to the aforementioned experimental studies, the Young’s modulus and Poisson’s ratio of the masonry and mortar utilized for the numerical simulations were 32,470 MPa and 19,850 MPa and 0.26 and 0.2, respectively.

Table 1 provides information on the material parameters of the masonry components, based on the data illustrated in Figure 8. In addition, the following parameters determining the shape of the plasticity surface were assumed for the masonry units (brick units and mortar joints): ∈ (eccentricity) = 0.1; ψ (dilation angle) = 30${}^{\circ }$; $\frac{\sigma_{b0}}{\sigma_{c0}}$ = 1.16; $K_{c}$ = 0.667; viscosity parameter = $10^{-5}$.

#### 2.3.2. Constitutive Model for SMA

**Figure 9:**
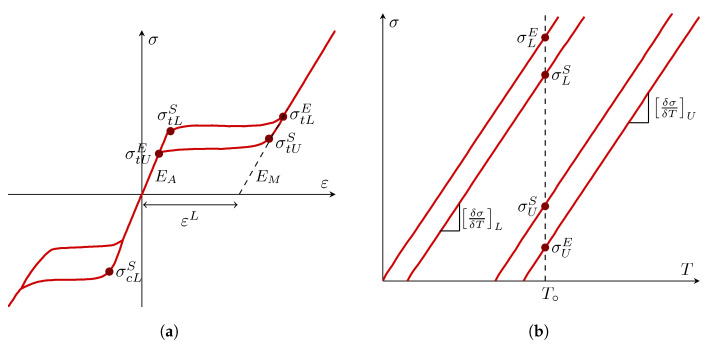
(**a**) Uniaxial response of a superelastic material and (**b**) stress–temperature curve of a superelastic material.

The pseudoelastic concept is based on the phase transformation of materials’ uniaxial hysteresis behavior (Figure 9a). Elastic characteristics were derived throughout the phase transition from the elastic modulus of austenite and martensite using the following equations [36,37]:

(3) $$E=E_{A}+\zeta \left(E_{M}-E_{A}\right)$$

(4) $$\nu =\nu_{A}+\zeta \left(\nu_{M}-\nu_{A}\right)$$

where ζ indicates the martensite proportion, $E_{A}$ stands for the austenite Young’s modulus, $E_{M}$ represents the martensite Young’s modulus, $\nu_{A}$ indicates the austenite Poisson’s ratio, and $\nu_{M}$ is the martensite Poisson’s ratio.

The cumulative strain increment is calculated as follows:

(5) $$\Delta \varepsilon =\Delta \varepsilon^{el}+\Delta \varepsilon^{tr}$$

where $\Delta \varepsilon^{tr}=\Delta \zeta \frac{\partial G^{tr}}{\partial \sigma }$ and $G^{tr}=q-p\tan\psi$, with $p=-\frac{1}{3}$ and $q=\sqrt{\frac{3}{2}S:S}$.

The stress rates at which the transition occurs considering the surrounding temperature can is specified by $T_{o}$. As illustrated in Figure 9b, these stress levels are considered to fluctuate exponentially with temperature.

According to the literature, Ni-Ti has superior hysteresis and energy dissipation qualities; however, due to relatively high prices, alternative types of SMAs, such as Cu-based and Fe-based SMAs, have been investigated for use in civil engineering applications as well [38]. As a result, in this work the stripes employed as retrofitting components on the masonry prism were made from two distinct SMAs (Ni-Ti and Cu-Zn-Al). The material properties were determined via laboratory testing, and the values were calibrated for use in the Abaqus software.

**Figure 10:**
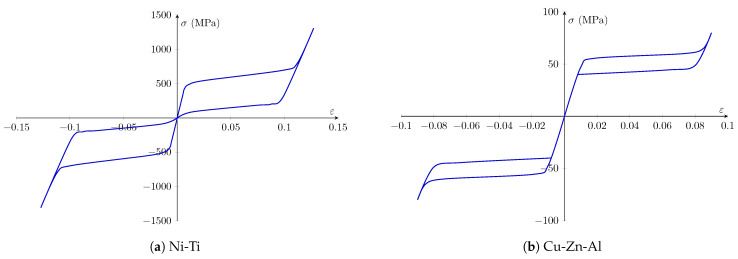
Numerical stress–strain curves of the SMA materials used in this study: (**a**) Ni-Ti and (**b**) Cu-Zn-Al.

The material characterization examinations and computational calculations for Ni-Ti are described in [39], while the details for Cu-Zn-Al are described in [40,41]. The Poisson ratio, Young’s modulus, and other variables relevant to hysteresis behavior of SMAs were determined for both Ni-Ti and Cu-Zn-Al; the data are displayed as a stress–strain curve in Figure 10. Based on the exploratory studies used to develop the computational models, the Young’s modulus of Ni-Ti in the austenite and martensite stages was 64,647 MPa and 21,000 MPa, respectively, and for Cu-Zn-Al it was 28,125 MPa and 16,000 MPa, respectively, with a Poisson’s ratio of 0.33 for both materials and phases.

Table 2 provides information on the material parameters of the SMAs (Ni-Ti and Cu-Zn-Al) based on the data illustrated in Figure 10.

### 2.4. Mesh Sensitivity Analysis

Mesh sizes of 40, 20, 10, 5, and 2.5 mm with hexahedral eight-node linear brick with reduced integration and hourglass control (C3D8R) element type were used to examine the mesh responsiveness of the simulations. The peak load in each model was computed in order to compare the findings from the numerical analysis with the results of experimental testing to analyze the mesh dependency of the models and validate the finite element analysis.

**Figure 11:**
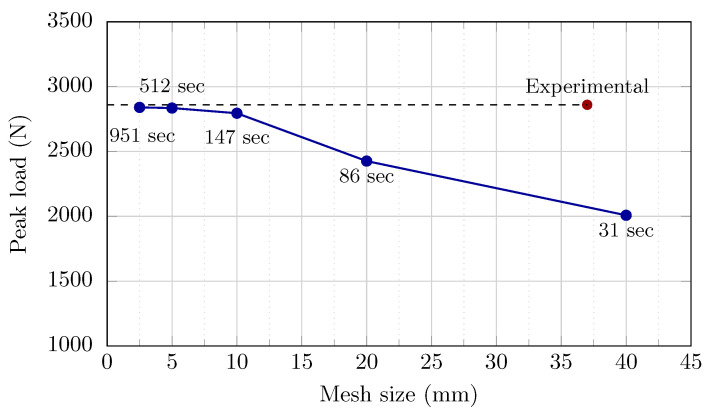
Peak load and run time comparison between the FEA results derived by increasing the mesh size and the peak load acquired from experimental tests.

Figure 11 depicts the outcome of the mesh convergence analysis. The investigation found that choosing a fine mesh size greater than or equivalent to 40 mm makes achieving convergence nearly impossible. The findings were unsatisfactory because of high inaccuracy and lack of convergence after crude mesh refining. Fine mesh sizes (2.5, 5, and 10 mm) produced good convergent outputs with a computed apex load close to the empirical data. Because Abaqus only assigns storage as demanded throughout processing, a larger amount of storage was required for calculations when employing lower mesh sizes. When the mesh size was decreased from 10 to 2.5 mm, for example, the RAM capacity almost tripled, from 4.2 GB to 13.9 GB. This suggests that an excessively fine mesh necessitates a considerable volume of RAM storage and lengthy execution duration, particularly for nonlinear analysis. As a result, the optimal element size for the proportion of precision, duration, and cost is 10 mm. Utilizing a computer system powered by an AMD^®^ Ryzen™7-4800 CPU@ 2.90 GHz and 32 GB RAM, the calculation duration with these particle sizes was around 147 s and 951 s, respectively, with 98% precision.

**Figure 12:**
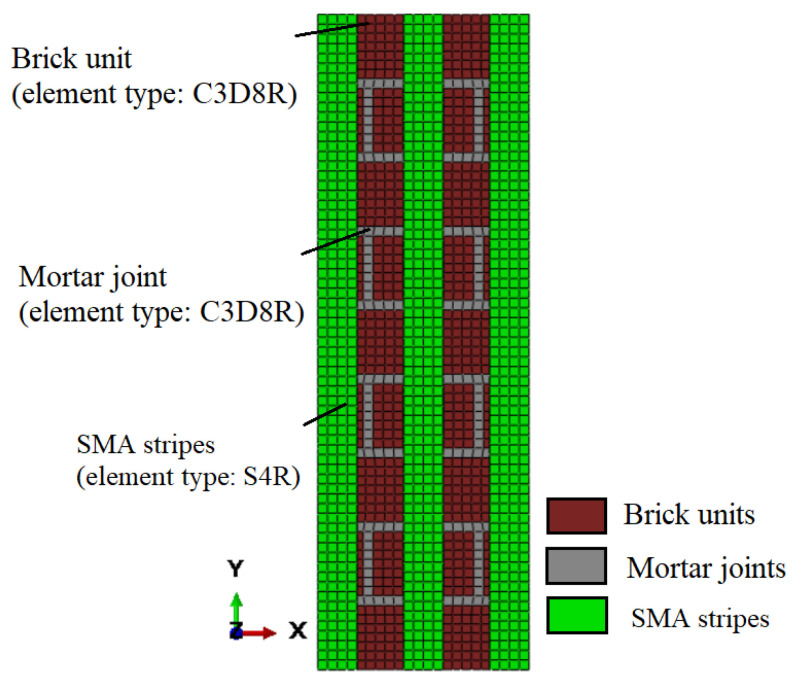
Schematic representation of retrofitted masonry prism with different types of FEs and materials.

The mesh convergence study resulted in a mesh size of 10 mm being adopted for the brick units and mortar joints for subsequent computational studies. The URM and retrofitting masonry prism models were made up of three parts: brick units, mortar joints, and SMA stripes. As illustrated in Figure 12, each of these parts was represented as a distinct component, then combined and meshed. Brick units and mortar joints were represented as eight-node deformable brick elements with reduced integration (C3D8R). A four-node quadrilateral shell element with decreased integration and a large-strain formulation was used to represent the SMA stripes (S4R).

### 2.5. Finite Element Model Validation

**Figure 13:**
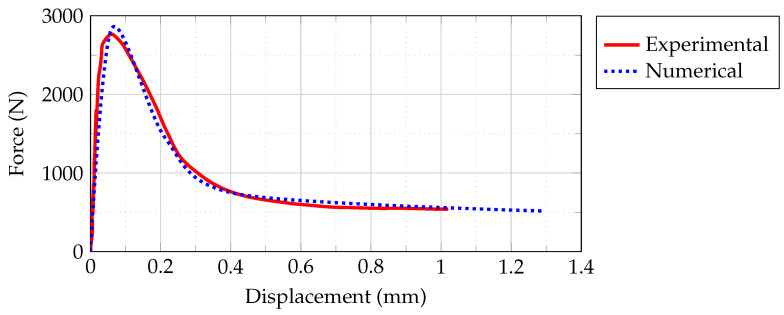
Comparison of empirical data obtained by [25] and computational lateral out-of-plane load-displacement curves.

The computational and empirical lateral out-of-plane load-displacement plots for the masonry prism are shown in Figure 13. The contrast demonstrates that the computational approach appropriately matches the response of the test samples. As noted throughout the experiment, the maximal load arises for insignificant amounts of dislocation linked with significant stiffness attributes of the specimen. The weakening division of the graph correlates well with the hard data, indicating that the computer simulation is able to represent the empirical characteristics. The maximum force and associated dislocation of the samples at failure were calculated within a 2–5% error of the experimental values (Table 3).

## 3. Finite Element Analysis Results

Further calculations were performed after the model was validated and an acceptable match between numerical and experimental testing was obtained. To explore the effect of increasing the thickness of the stripes as well as of using different types of SMA material (Ni-Ti and Cu-Zn-Al), different thickness values and SMA materials (Ni-Ti and Cu-Zn-Al) were employed.

It is significant to note that due to the relatively low loading quantities used in this investigation the retrofitting strips were elastic and functioned the same as any other conventional metals due to strain compatibility between the SMA and masonry prism. As a result, greater loads were applied to achieve good strain compatibility between the masonry prism and SMA retrofitting sections and to benefit from the pseudoelastic feature of SMAs.

**Figure 14:**
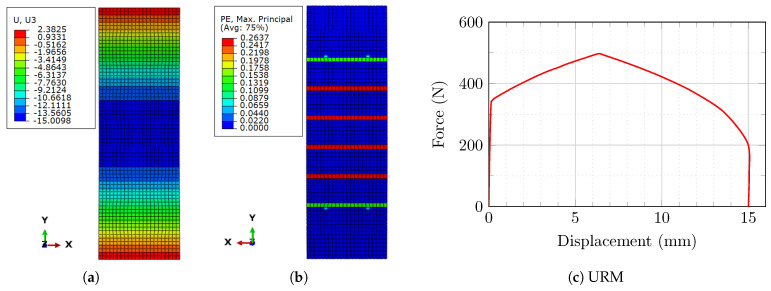
The behavior of the masonry prism: (**a**) residual displacement in the direction of loading (U_3_, U${}_{Z}$ or out-of-plane); (**b**) residual plastic strains; (**c**) relationship between reaction forces and maximum displacement.

After the unloading phase, remnant deformations were seen practically everywhere on the masonry prism (excluding where the boundary condition supports were placed). The maximum residual displacement was approximately 15 mm in the orientation of the imposed pressure (Figure 14a).

Plastic strains were another key component in the deterioration of the brickwork structure. Six parallel ridges of mortar in the middle of the masonry prism (on the opposing side where the loading was applied) showed significant values of plastic strains due to the weaker characteristics of the mortar (particularly in the tension domain) (Figure 14b).

The relationship between reaction forces and mid-span displacement in the masonry prism during the loading–unloading method is depicted in Figure 14c. It can be observed that the wallonly restores a minimal percentage of its deformations when unloading is complete.

**Figure 15:**
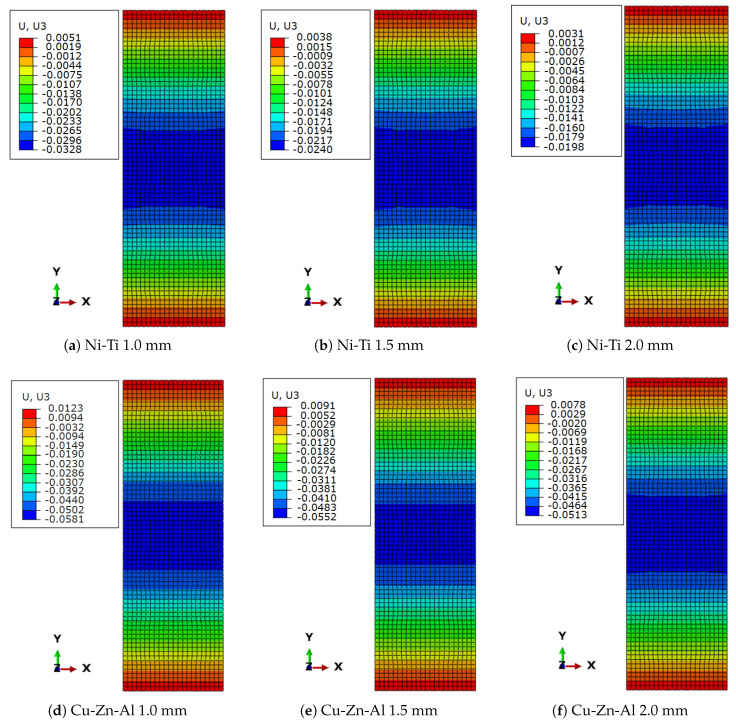
Residual displacement in the direction of loading (U_3_, U${}_{Z}$ or out-of-plane) in retrofitted models: (**a**) 1.0 mm Ni-Ti; (**b**) 1.5 mm Ni-Ti; (**c**) 2.0 mm Ni-Ti; (**d**) 1.0 mm Cu-Zn-Al; (**e**) 1.5 mm Cu-Zn-Al; (**f**) 2.0 mm Cu-Zn-Al.

Figure 15 depicts the residual displacement in models where the masonry prism was retrofitted with SMA stripes. The residual displacement was greatly decreased in all models compared to the masonry prism model without any retrofitting feature. The masonry prism reinforced with Ni-Ti stripes performed better in terms of residual displacement reduction than the masonry prism reinforced with Cu-Zn-Al SMA; moreover, the impact of increasing the thickness of stripes was more significant for Ni-Ti than for Cu-Zn-Al. The difference in residual displacement reduction from 1 mm to 2 mm thickness was 40% in Ni-Ti and 11% in Cu-Zn-Al.

**Figure 16:**
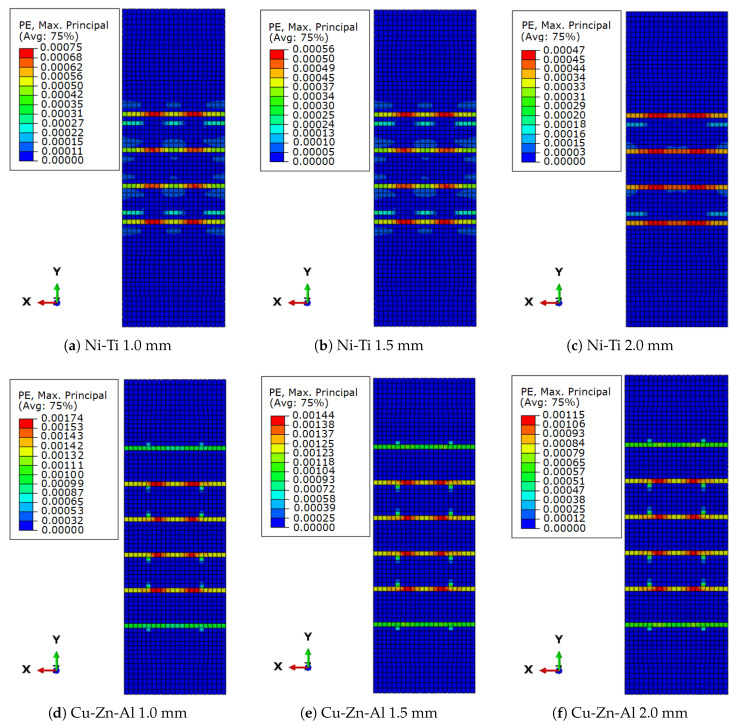
Plastic residual strain in retrofitted models: (**a**) 1.0 mm Ni-Ti; (**b**) 1.5 mm Ni-Ti; (**c**) 2.0 mm Ni-Ti; (**d**) 1.0 mm Cu-Zn-Al; (**e**) 1.5 mm Cu-Zn-Al; (**f**) 2.0 mm Cu-Zn-Al.

Figure 16 depicts the residual plastic strains in brickwork prism models reinforced with SMA strips. When compared to the masonry prism model with no retrofitting element, all models had much lower plastic strains. In terms of plastic strain decrease, the masonry prism reinforced with Ni-Ti stripes outperformed the masonry prism reinforced with Cu-Zn-Al SMA; furthermore, increasing the thickness of the stripes had a greater influence on Ni-Ti than on Cu-Zn-Al. For Ni-Ti, the difference when lowering residual displacement from 1 mm to 2 mm thickness was 42%, whereas for Cu-Zn-Al it was 35%.

Although the pseudoelastic property of SMA allows for substantial energy dissipation and a resulting reduction in residual stresses, one important aspect should be noted here. Even when higher loads were applied, the SMA material cannot entirely recover to return the wall to its original configuration. While not addressed in this research, we speculate that full recovery might be achieved by assuring full strain compatibility between the SMA and the masonry prism. This might be done by material tuning, the use of stronger masonry material, or coupling of the SMA with other retrofitting components such as Fiber Reinforced Polymers (FRPs) and Engineered Cementitious Composite (ECC), as well as through other feasible options.

**Figure 17:**
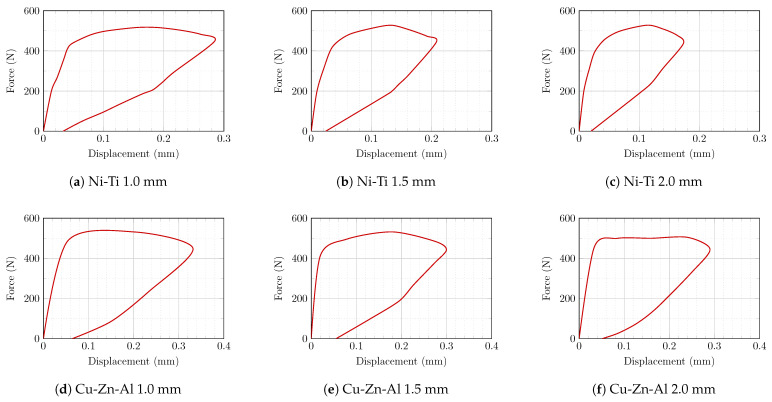
Relationship between reaction force at the support and mid-span displacement in retrofitted models: (**a**) 1.0 mm Ni-Ti; (**b**) 1.5 mm Ni-Ti; (**c**) 2.0 mm Ni-Ti; (**d**) 1.0 mm Cu-Zn-Al; (**e**) 1.5 mm Cu-Zn-Al; (**f**) 2.0 mm Cu-Zn-Al.

Figure 17 demonstrates the relationship between reaction forces and maximum displacement in the SMA-retrofitted models. Compared to the models with Cu-Zn-Al SMA stripes, the masonry prism strengthened with Ni-Ti SMA stripes showed lower mid-span and residual displacement despite having almost equal maximum reaction forces. In all cases, increasing the thickness lowered the maximum displacement while having no influence on the ultimate residual displacement.

Table 4 summarizes the numerical findings of the masonry prisms with and without retrofitting components. Both retrofitting materials (Ni-Ti and Cu-Zn-Al) demonstrated good recovery in terms of lower residual displacement compared to the masonry prism without any reinforcing element. The URM prism had a very low recovery of only 0.4%. Models with Ni-Ti stripes, on the other hand, recovered at a rate of around 88%, while models with Cu-Zn-Al recovered at a rate ranging from 80% to 82.4%. Increasing the thickness of the SMA stripes had relatively little influence on the models’ recovery rates.

## 4. Conclusions

This research explores the computational modeling of masonry prisms retrofitted with two SMA materials (Ni-Ti and Cu-Zn-Al) and stripes with variable thickness (1.0 mm, 1.5 mm, and 2.0 mm) exposed to out-of-plane loading. The material parameters used in this investigation had earlier been experimentally evaluated and analytically verified. It is important to note that in order to keep this paper brief only a few effects were assessed in this study; however, it would be advantageous to further investigate this topic while controlling the size dependency by increasing the prism dimensions (up to a real-scale masonry wall), the effect of impact loading on the masonry panels with SMA, strain compatibility between SMA and masonry material, etc. In general, the following conclusions can be drawn:

- Because of the high stiffness of the SMA material, the SMA-reinforcing stripes reduced the maximum displacement in the masonry prisms from 15.06 mm to 0.17–0.33, depending on the SMA material and thickness.
- Thanks to the high stiffness and re-centering capabilities of SMA materials, the masonry prism’s residual displacement was reduced from 15.0 mm to 0.02 mm–0.06 mm, depending on the SMA material and thickness.
- Stripes formed from Ni-Ti SMA performed better than those made of Cu-Zn-Al in terms of improving masonry prism performance and lowering the damage incurred. Furthermore, increasing the thickness of the stripes was somewhat more efficient in Ni-Ti SMA stripes than in Cu-Zn-Al stripes.
- Although the SMA material used in the retrofitting strips greatly mitigated the damage to the masonry prism with good effect, recovery was not total. This issue could be related to strain interoperability and the large difference in stiffness between SMA and brittle materials such as brick and mortar. In order to appropriately address the issue, this might be a potential subject of future research.

## Figures and Tables

**Table 1:** Yield stress and corresponding strain values of brick units and mortar joints [34].

Brick units			
Compression stiffening properties		Tension stiffening properties	
Yield stress (MPa)	Inelastic strain	Yield stress (MPa)	Cracking strain
26.12	0	5.93	0
68.16	0.0081	4.74	0.0002
77.89	0.0114	3.52	0.0005
84.54	0.0144	2.04	0.0009
87.78	0.0187	0.90	0.0020
72.33	0.0313	0.52	0.0030
36.78	0.0352	0.23	0.0053
18.85	0.0403	-	-
10.66	0.0460	-	-
**Mortar joints**			
Compression stiffening properties		Tension stiffening properties	
Yield stress (MPa)	Inelastic strain	Yield stress (MPa)	Cracking strain
1.74	0	1.11	0
3.10	0.0008	0.83	0.0009
4.99	0.0032	0.48	0.0030
5.84	0.0048	0.25	0.0058
6.51	0.0069	0.16	0.0079
6.98	0.0095	-	-
7.07	0.0120	-	-
5.72	0.0242	-	-
3.48	0.0348	-	-

**Table 2:** Properties of Ni-Ti and Cu-Zn-Al SMAs used in this study.

Property	Unit	Ni-Ti [39]	Cu-Zn-Al [40,41]
$E_{A}$	Mpa	64,647	21,000
$E_{M}$	Mpa	28,125	16,000
$\nu_{A}$	-	0.33	0.33
$\nu_{M}$	-	0.33	0.33
$\varepsilon^{L}$	%	9.0	7.5
$\sigma_{tL}^{S}$	Mpa	450	55
$\sigma_{tL}^{E}$	Mpa	700	60
$\sigma_{tU}^{S}$	Mpa	200	45
$\sigma_{tU}^{E}$	Mpa	30	40
$\sigma_{cL}^{S}$	Mpa	450	55

**Table 3:** Computational model and experimental test outcomes for masonry prism.

	Experimental [25]	Numerical	Difference (%)
Peak load (N)	2860	2795	2.27
Displacement at peak load (mm)	0.058	0.055	5.00

**Table 4:** Summary of the FEA results.

	Max. Plastic Strain	Max. Displacement (mm)	Residual Displacement (mm)	Recovery (%)
URM	0.2637	15.06	15.000	0.40
URM with Ni-Ti 1.0 mm	0.0007	0.29	0.033	88.62
URM with Ni-Ti 1.5 mm	0.0005	0.20	0.024	88.00
URM with Ni-Ti 2.0 mm	0.0004	0.17	0.020	88.23
URM with Cu-Zn-Al 1.0 mm	0.0017	0.33	0.064	80.06
URM with Cu-Zn-Al 1.5 mm	0.0014	0.30	0.055	81.60
URM with Cu-Zn-Al 2.0 mm	0.0011	0.29	0.051	82.40

## Data Availability

The data presented in this article are available upon request to the corresponding authors.
